# Techniques to measure cardiac output: minimally invasive method versus thermodilution

**DOI:** 10.1186/cc10823

**Published:** 2012-03-20

**Authors:** A Donati, S Tondi, A Carsetti, R Domizi, C Melia, D Kolgjini, C Munch, C Scorcella, P Pelaia

**Affiliations:** 1 Università Politecnica delle Marche, Ancona, Italy

## Introduction

Hemodynamic monitoring is important to manage critically ill patients. The thermodilution pulmonary catheter is considered the gold standard; however, it is invasive and associated with the onset of complications. Our study compared cardiac output (CO) obtained with the MostCare (COMC), which uses the pressure recording analytical method, to CO obtained with a Swan-Ganz (COSG) catheter in hemodynamically unstable patients.

## Methods

We conducted a prospective clinical study in our cardiosurgical ICU. Sixteen post-cardiosurgical adult patients were enrolled. They had a Swan-Ganz catheter and were mechanically ventilated. The Swan-Ganz catheter was connected to the monitor Vigilance Edwards^®^, while the MostCare was connected to the patient's artery. For each patient three measurements of CO have been carried out and the mean was considered for statistical analysis. The correlation coefficient, Bland-Altman test and percentage of error were measured.

## Results

The correlation coefficient between COSG and COMC was 0.824 (0.567 to 0.935, 95% CI; *P *< 0.001) The Bland-Altman analysis showed a mean difference between the two methods (bias) of 0.22 ± 0.55 l/minute/m^2 ^with lower and upper 95% limits of confidence of -0.87 and 1.30 l/minute/m^2 ^respectively. The percentage of error was of 25%.

## Conclusion

This study demonstrated a good correlation between the two methods. MostCare is resulted to be reliable and accurate even in hemodynamically unstable patients. It would be interesting to study the new device before and after having modified the therapy, such as fluid challenge or inotropic therapy or the use of vasopressors.
